# A Noninvasive Prediction Model for Hepatitis B Virus Disease in Patients with HIV: Based on the Population of Jiangsu, China

**DOI:** 10.1155/2021/6696041

**Published:** 2021-03-29

**Authors:** Yi Yin, Mingyue Xue, Lingen Shi, Tao Qiu, Derun Xia, Gengfeng Fu, Zhihang Peng

**Affiliations:** ^1^Department of Epidemiology and Biostatistics, School of Public Health, Nanjing Medical University, Nanjing, Jiangsu 211166, China; ^2^Hospital of Traditional Chinese Medicine Affiliated to the Fourth Clinical Medical College of Xinjiang Medical University, Urumqi, China; ^3^Institute of HIV/AIDS/STI Prevention and Control, Jiangsu Provincial Center for Diseases Control and Prevention, Nanjing, Jiangsu 210009, China

## Abstract

**Objective:**

To establish a machine learning model for identifying patients coinfected with hepatitis B virus (HBV) and human immunodeficiency virus (HIV) through two sexual transmission routes in Jiangsu, China.

**Methods:**

A total of 14197 HIV cases transmitted by homosexual and heterosexual routes were recruited. After data processing, 12469 cases (HIV and HBV, 1033; HIV, 11436) were left for further analysis, including 7849 cases with homosexual transmission and 4620 cases with heterosexual transmission. Univariate logistic regression was used to select variables with significant *P* value and odds ratio for multivariable analysis. In homosexual transmission and heterosexual transmission groups, 10 and 6 variables were selected, respectively. For identifying HIV individuals coinfected with HBV, a machine learning model was constructed with four algorithms, including Decision Tree, Random Forest, AdaBoost with decision tree (AdaBoost), and extreme gradient boosting decision tree (XGBoost). The detective value of each variable was calculated using the optimal machine learning algorithm.

**Results:**

AdaBoost algorithm showed the highest efficiency in both transmission groups (homosexual transmission group: accuracy = 0.928, precision = 0.915, recall = 0.944, *F* − 1 = 0.930, and AUC = 0.96; heterosexual transmission group: accuracy = 0.892, precision = 0.881, recall = 0.905, *F* − 1 = 0.893, and AUC = 0.98). Calculated by AdaBoost algorithm, the detective value of PLA was the highest in homosexual transmission group, followed by CR, AST, HB, ALT, TBIL, leucocyte, age, marital status, and treatment condition; in the heterosexual transmission group, the detective value of PLA was the highest (consistent with the condition in the homosexual group), followed by ALT, AST, TBIL, leucocyte, and symptom severity.

**Conclusions:**

The univariate logistics regression combined with the AdaBoost algorithm could accurately screen the risk factors of HBV in HIV coinfection without invasive testing. Further studies are needed to evaluate the utility and feasibility of this model in various settings.

## 1. Background

Acquired immune deficiency syndrome (AIDS) caused by human immunodeficiency virus (HIV) is a global health crisis [1]. HIV invades T lymphocytes (CD4+) cells of the human immune system, leading to a variety of opportunistic infections and death [2, 3]. Hepatitis B (HB), a chronic disease characterized by hepatitis B virus (HBV) infection for more than 6 months and varying degrees of inflammatory necrosis/fibrosis of the liver, also poses a global health threat [4]. Worldwide, estimates suggest that more than 2 billion people have been infected with HBV, including 248 million chronical infections (defined as hepatitis B surface antigen (HBsAg) positivity) [4, 5]. Approximately 10% of HIV-infected people are also chronically coinfected with HBV [6]. Coinfected patients present with rapidly progressive liver disease, facing a higher risk of cirrhosis and even death [7]. HBV infection worsens the prognosis in HIV-positive people [6]. Therefore, efficient tools should be developed to identify HIV/HBV coinfection before the establishment of specific treatments.

Considering China's greatest HBV population worldwide, it is supposed that the HIV/HBV coinfection rate may exceed the global average [8]. HIV and HBV share similar transmission routes and risk factors, making it difficult to create an accurate method for early distinguishment of HBV from HIV infection [8].

It is difficult to use traditional logistic regression to process demographic and serological data which are often nonlinear, abnormal, and heterogeneous [9]. But machining learning (ML) provides a chance. Compared to the logistic regression model, ML analysis does not require data structure, since ML can balance the deviation and variance of data. Nowadays, ML methods have wide applications in the medical field, such as diagnosis of cancers, diabetes, medical imaging, and pediatric diseases [10–12]. In the present study, we established a ML model for identifying HIV patients coinfected HIV with HBV patients based on four algorithms.

Previous studies have explored the differences in baseline variables between HIV patients and HIV/HBV coinfected patients. Compared to HBV infection group, the HIV/HBV coinfection group had a lower level of platelets [13, 14] and a higher level of liver enzymes, especially aspartate aminotransferase and alanine aminotransferase [15–17]. At the onset of coinfection, CD4 count dropped significantly and recovered slowly after cART [16, 18, 19]. Besides, compared to HIV infection, HBV/HIV coinfection raised serum bilirubin levels [17]. Nevertheless, HBV/HIV coinfection could perplex the diagnosis of HBV on account of spontaneous loss of hepatitis B surface antibody and reactivation of HBV replication [14]. Hepatitis B surface antigen (HBsAg) seroclearance has been analyzed during the treatment and prognosis of CHB using four ML algorithms, including extreme gradient boosting (XGBoost), Random Forest, Decision Tree, and logistic regression (LR) [20]. However, our study is the first to use them to screen patients with HBV/HIV coinfection.

## 2. Methods

### 2.1. Study Population

Recruited were 14197 HIV and HIV and HBV cases infected through homosexual transmission and heterosexual transmission recorded between 2005 and 2019. Demographic and serological data were obtained from Jiangsu Provincial Central of Disease Control (CDC). Demographic information mainly consisted of age, marital status, severity of symptoms (fever, cough, and so on), clinical stage, weight, and height. Four clinical stages of AIDS were included. The geographic setting was divided into Jiangsu province and other provinces. The marital status was divided into four classes: unmarried, married or cohabiting, divorced or separated, and widowed. Next, “yes” or “no” was used to describe the presence/absence of tuberculosis, drug use, and symptoms. The serological indexes contained blood creatinine level (CR), leucocyte, triglyceride (TG), total cholesterol (TC), aspartate aminotransferase (AST), alanine aminotransferase (ALT), and total bilirubin (TBIL).

### 2.2. Data Processing

A total of 14197 HIV and HIV/HBV cases were recruited from the CDC of Jiangsu. Variables with missed and abnormal values, as well as samples with many null variables (totally 4 variables and 1728 cases), were deleted. Finally, 12469 cases (1033 HIV and HBV cases and 11436 HIV cases) left were used for further analysis, including 7849 infected through homosexual transmission and 4620 through heterosexual transmission. And for some variables with few nulls, we replaced them with the most frequent values or the mean number for continuous variables. Categorical variables were presented as the frequency number (percentage), and continuous variables in a normal distribution were presented as mean ± standard deviation.

### 2.3. ML Model Establishment

A ML model was established based on Decision Tree, Random Forests, AdaBoost, and XGBoost algorithms. Specific, Decision Tree is a supervised learning method based on a cluster of “if-then” regulations to increase readability [21]. However, Decision Tree may present with big variance and overfitting. To solve this, we employed different trees, like Random Forests, to improve the prediction ability of the model. AdaBoost and XGBoost algorithms were used to combine stumps of trees [1]. AdaBoost is based on gradient, and XGBoost is based on the weight of data with the wrong classification. In this study, we used these four methods for the classification of HBV patients.

### 2.4. Feature Selection

Characteristics of specific value can be selected using ML and traditional statistical methods, such as Random Forests [22], logistic regression [23], principal component analysis(PCA), analysis of variance, and Fisher discriminant rate [24]. In this study, we used univariate logistics regression to keep the original data structure and make the variables comprehensible. Corresponding odds rate (OR) with 95% confident interval (95% CI) and *P* value were calculated.

### 2.5. Data Balancing

In HIV patients, the number of patients coinfected with HBV was imbalanced. Generally, it is more difficult to classify a small population than a large population [25]. Therefore, the data were balanced with random undersampling (RUS) and random oversampling (ROS). ROS was used to remove the fraction of HIV/AIDS patients without HBV, then balance the numbers in both categories. After ROS, the computing speed and memory are increased, but some information may be lost. In this study, Synthetic Minority Oversampling Technique (SMOTE), a method evolving from ROS, was used for data balance. SMOTE could increase the number in the smaller class through some regulations. For example, disturbing data and random noise might be added to achieve class balance, meanwhile saving the original information.

### 2.6. Model Evaluation

After balancing, the data were divided into two groups: development group (70%) and validation group (30%) [26]. In order to improve the accuracy of Decision Tree, we plotted the “verification curve” based on the fivefold cross verification. The receiver operating characteristic curve (ROC) [27], confusion matrix, accuracy rate, precious rate, recall rate, and comprehensive evaluation index (*F*-Measure) were used to judge the accuracy of the model. Relative concepts were showed as follows.

Confusion matrix presents the diagnostic results in the form of tables, summarizes the number of correct and incorrect classifications, and divides them into categories. The confusion matrix shows which part of the classification model will be obfuscated during the identification, and the tabular form of output is shown in Table 1.

Accuracy refers to the proportion of correct classification, calculated with the following formula:

(1) $$\begin{array}{c} \text{Accuracy}=\frac{\text{TP}+\text{TN}}{\text{TP}+\text{TN}+\text{FP}+\text{FN}}. \end{array}$$

However, in dealing with imbalanced data, it is difficult to express the accuracy of the classifier. For example, 100 cases in the development group, the positive results accounted for 99%. Even if all cases are predicted to be positive and the accuracy of the model is more than 90%, the result makes no sense.

Precision means the proportion of all correctly predicted HIV coinfected with HBV cases against all actual HIV coinfected with HIBV cases. It represents the probability of a correct prediction among all the results:

(2) $$\begin{array}{c} \text{Precision}=\frac{\text{TP}}{\text{TP}+\text{FP}}. \end{array}$$

The difference between accuracy and precision is that the former represents the accuracy of prediction in positive samples, while the latter represents the accuracy of prediction in the total of positive and negative samples.

Recall represents the proportion of all correctly predicted cases to all predicted HIV coinfected with HBV cases.

(3) $$\begin{array}{c} \text{Recall}=\frac{\text{TP}}{\text{TP}+\text{FN}}. \end{array}$$

It is a trade-off measurement with precision and should be balanced according to actual requirements.

*F*-Measure is the weighted harmonic average of Precision and Recall.

(4) $$\begin{array}{c} F‐1=\frac{2\times \text{Precision}\times \text{Recall}}{\text{Precision}+\text{Recall}}. \end{array}$$

ROC curve is used for evaluating the performance of the ML model. The vertical coordinate of the ROC curve is the true rate (TPR), and the horizontal coordinate is the false-positive rate (FPR).

AUC curve represents the area under the ROC, whose value is between 0.5 and 1. The value of AUC close to 1 indicates the better performance of the model [28].

All data analyses were carried out with R software 4.0.2 version and Python software 3.7 version.

## 3. Results

**Figure 1:**
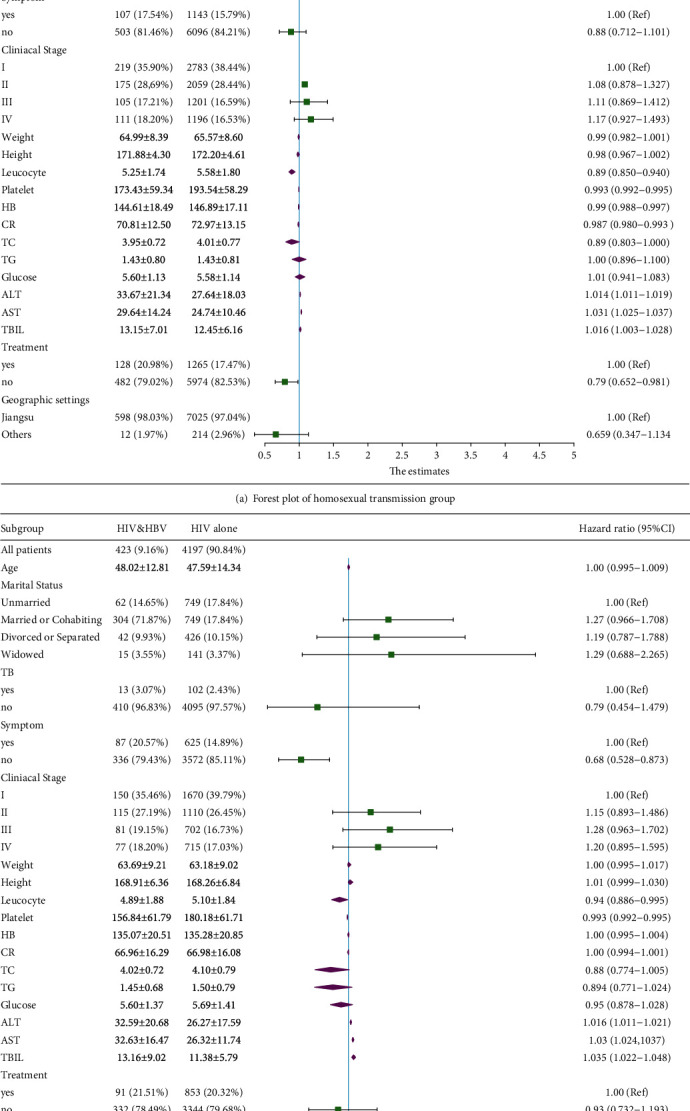
Forest plots of univariate logistic regression analysis in both groups.

After data processing, 12469 individuals were incorporated into the model. There were 7849 cases in the homosexual transmission group, including 7239 HIV cases and 610 HIV/HBV coinfected cases. There were 4620 samples in the heterosexual transmission group, including 4197 HIV cases and 423 HIV and HBV cases. Univariate logistic regression analysis was performed in two groups. In the homosexual group, the risk of HBV infection increased with age. Compared with the unmarried, the cohabitors and the divorced or separated had a higher risk of HBV. Some blood indexes, such as leucocyte, platelet, blood creatinine, ALT, AST, and TB, were significantly different between HIV and HIV and HBV groups, suggesting that they can be used as risk-evaluating indicators. In the heterosexual transmission group, AST, ALT, TB, and platelet levels were significantly different between the HIV and HIV and HBV groups, which was consistent with that of the homosexual group. Compared with the unmarried, other populations showed no differences in these indicators, which is different from the condition in the homosexual group. In both groups, the geographic setting had no differences between HIV and HIV and HBV groups. Details were shown in Table 2. Moreover, we drew the forest plots of the results in univariate logistic regression (Figure 1). Figure 1 depicted the baseline information of the HIV and HIV and HBV cases and the OR values with 95% CI in homosexual and heterosexual transmission groups.

The original data in the heterosexual transmission group contained 4620 cases. After calculation with SMOTE algorithm, the number was raised to 8385 cases that were randomly divided into the development group (5870 cases) and validation group (2515 cases). In the homosexual transmission group, the original data consisted of 7849 cases. After calculation with SMOTE algorithm, the number was raised to 14482 cases that were randomly divided into the development group (10137 samples) and validation group (4345 samples). The ratio of the numbers in development group to validation group was 7 : 3. Results of the SMOTE algorithm were presented in Table 3.

**Figure 2:**
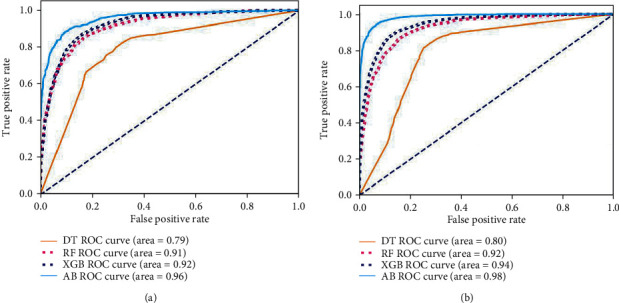
ROC curve of all algorithms in homosexual group and heterosexual group. (a) Homosexual group; (b) heterosexual group. DT: decision tree; RF: random forest; AB: AdaBoost; XGB: XGBoost.

The performances of ML model in the heterosexual and homosexual groups were shown in Table 4 and Table 5. The confusion matrix was shown by the heat map. The larger the value, the darker region. The color of TN and TP was almost orange. On the contrary, the lighter FN and FP regions, the higher accuracy of the model. We found that AdaBoost algorithm was the most accurate in both groups (homosexual group: accuracy = 0.928, precision = 0.915, recall = 0.944, *F* − 1 = 0.930, and AUC = 0.96; heterosexual group: accuracy = 0.892, precision = 0.881, recall = 0.905, *F* − 1 = 0.893, and AUC = 0.98). ROC curves of four algorithms in homosexual and heterosexual groups were shown in Figure 2.

**Figure 3:**
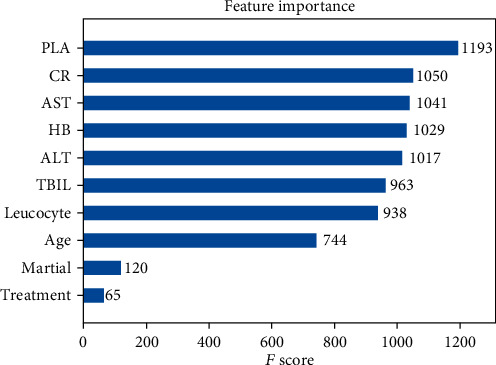
Detective values (*F* score) calculated by the AdaBoost model in homosexual group.

**Figure 4:**
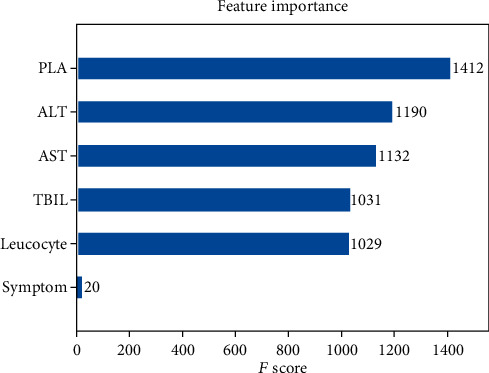
Detective values (*F* score) calculated by the AdaBoost model in heterosexual group.

The AdaBoost algorithm had the strongest capacity in classification. Therefore, we sorted out the significant variables using AdaBoost in homosexual and heterosexual groups. AdaBoost calculated the detective value of each variable with three algorithms. However, the three scores of each variable showed no significant difference, indicating their mild effect on the rank level. Finally, we chose the default method for further analysis. In the homosexual transmission group, 10 variables were selected by univariate logistic regression. Among them, the detective value of PLA was the highest, followed by CR, AST, HB, ALT, TBIL, leucocyte, age, marital status, and treatment condition (Figure 3).  Figure 4 depicted the scores of the 6 variables selected from univariate logistic regression in the heterosexual group. Among them, the score of PLA was the highest (consistent with the condition in the homosexual group), followed by ALT, AST, TBIL, leucocyte, and symptom severity.

## 4. Discussion

Globally, among the annual 1.3 million HIV-related deaths, 96% are caused by complications of chronic hepatitis, 66% by HBV [29]. In China, about 8.7% of HIV/AIDS patients are coinfected with HBV that shares the same transmission routes [30]. By 31 October 2019, the prevalence of AIDS had maintained at a low level, but HIV positive rate is still high in some high-risk groups. Data show that since the “thirteenth Five-Year” (2016-2020), the HIV positive rate of MSM stays between 6.97% and 8.58%. AIDS is mainly transmitted through sexual routes. In a large study of HIV-positive Chinese, the prevalence of HBV and HIV coinfection was 9.5%, highest in Eastern China (14.5%) and lowest in Central China (5.0%) [31]. Over one-third of HIV and HBV coinfected patients develop moderate-to-severe liver disease [32]. However, it is difficult to find an accurate model for classifying patients coinfected with HBV in HIV.

In the present study, a ML model was constructed with four algorithms. The accuracy, recall, precision, and AUC value of each algorithm were analyzed. SMOTE method was used to balance the data. In the homosexual transmission group, the accuracy calculated by DT, RF, AdaBoost, and XGBoost was 0.779, 0.844, 0.928, and 0.875, respectively; the precision was 0.750, 0.804, 0.915, and 0.844, respectively; the recall was 0.839, 0.910, 0.944, and 0.919, respectively; the AUC was 0.805, 0.921, 0.982, and 0.944, respectively; the value of *F*-1 was 0.792, 0.854, 0.930, and 0.880, respectively. In the heterosexual transmission group, the accuracy was 0.762, 0.837, 0.892, and 0.863, respectively; the precision was 0.710, 0.838, 0.881, and 0.860, respectively; the recall was 0.888, 0.836, 0.905, and 0.868, respectively; the AUC was 0.794, 0.911, 0.957, and 0.923, respectively; the value of *F*-1 was 0.789, 0.837, 0.893, and 0.864, respectively. These data supported that AdaBoost algorithm was the most accurate tool (homosexual group: accuracy = 0.928, precision = 0.915, recall = 0.944, *F* − 1 = 0.930, and AUC = 0.96; heterosexual group: accuracy = 0.892, precision = 0.881, recall = 0.905, *F* − 1 = 0.893, and AUC = 0.98).

We next chose the AdaBoost algorithm for further analysis. Among 10 variables selected by univariate logistic regression in homosexual groups, through ML analysis, we calculated the importance sorting of these variables. The higher the feature score is, the more contribution a variable makes to the detective of coinfected with HBV patients in HIV cases. PLA was the most accurate variable in our study, closely followed by CR, AST, HB, ALT, TBIL, leucocyte, age, marital status, and treatment condition, which is similar to previous studies [33–35]. PLA had the highest detective value in the heterosexual group, followed by ALT, AST, TBIL, leucocyte, and symptom severity. Some studies have demonstrated a strong association of PLT, age, AST, ALT, and INR with liver fibrosis in coinfected patients [35]. AST-to-platelet ratio index (APRI) and FIB-4 score have been proposed for staging hepatic fibrosis [36]. It needs future study to explain the differences between two transmission groups.

Extensive studies have attested to the fact that HBV and HIV infections have mutually adverse effects. Compared to HIV patients, HIV and HBV coinfected patients display faster immunological and clinical progression, stronger hepatotoxicity after initiation cART, and higher morbidity and mortality. Similarly, HIV impairs host immunity against HBV, because CD4 cells are destructed by HIV. Coinfection with HIV can accelerate HBV-induced liver damage, leading to cirrhosis and advanced liver disease. Liver enzymes (ALT, AST mainly), synthetic function (albumin and prothrombin time), bilirubin, complete blood count, and platelet count, especially a gradual decline in platelet count, may be more sensitive markers of progressing liver fibrosis [37]. However, more sensitive markers should be explored.

In both the homosexual and heterosexual groups, there was a statistically lower platelet count and higher ALT and AST levels in HBV and HIV coinfected patients, indicating the presence of advanced fibrosis. The higher baseline serum bilirubin suggests that tuberculosis is more associated with HIV and HBV coinfection than HIV infection. Multiple factors contribute to the hematological manifestations of HIV disease, like anemia, neutropenia, and thrombocytopenia. This may explain why the leucocyte count was lower in the coinfected patients in both groups.

HIV infection influences all hematopoietic cell lineages and can cause a spectrum of hematological abnormalities [38]. The pathogenesis of HIV-associated anemia is not fully understood, but is assumed to be multifactorial [32]. Besides, we found an association between CD4 count and hemoglobin level: anemia rate significantly increased in patients with low CD4 count, and low hemoglobin level increased the risk of death in patients with AIDS, independent of the CD4 count [32]. In the homosexual transmission group showed a lower hemoglobin level, indicating that HBV facilitates HIV progression and increases the risk of death.

CR can be used to assess kidney function [39]. The study based on 90 newly diagnosed HIV patients with viral hepatitis infection in Cape Coast Teaching Hospital HIV clinic showed that severe kidney malfunction (chronic kidney disease stage 4, with eGFR < 15 mL/min/1.73 m^2^) was only and nearly significant in those with HIV1/HBV [31]. This finding is also supported by the higher CR in heterosexual coinfected patients in the present study.

It is a great pity that we did not collect the data of CD4+ T cell count, which is a reliable marker for assessing liver disease progression. Previous studies have shown that the baseline CD4 cell count is lower in HBV/HIV coinfected individuals compared to monoinfected individuals. This association may be related with HBV genotype, chronic immune activation, cytokines, and apoptotic pathways involved in these infections [14].

There are some limitations in our study as well. First, the data are provided by the CDC, so some potentially relevant variables may be left out. Second, other ML algorithms should be evaluated. Third, the efficiency of the model should be clinically validated.

## 5. Conclusions

The univariate logistics regression combined with the AdaBoost algorithm could accurately screen the risk factors of HBV in HIV coinfection without invasive testing. We found that AST was the most significant detective variable in both homosexual and heterosexual transmission groups, which should be paid more attention. Different sex transmission routes of HIV had different risk of coinfected with HBV, as well as risk factors, but detailed evidence required further studies. Further studies are needed to evaluate the utility and feasibility of this model in various settings.

## Figures and Tables

**Table 1:** Confusion matrix.

	HIV and HBV (prediction)	HIV (prediction)
HIV and HBV (actual)	TP	FN
HIV (actual)	FP	TN

**Table 2:** Basic characteristics of variables and univariate logistic analysis.

Homosexual					Heterosexual			
Variables	HIV *N* = 7239 (92.22%)	HIV and HBV *N* = 610 (7.78%8)	OR (95% CI)	*P* value	HIV *N* = 4197 (90.84%)	HIV and HBV *N* = 423 (9.16%)	OR (95% CI)	*P* value
Age (years)	37.43 ± 12.21	40.83 ± 10.89	1.022 (1.015, 1.028)	<0.01	47.59 ± 14.34	48.02 ± 12.81	1.00 (0.995, 1.009)	0.55
Marital status (%)								
Unmarried	3943 (54.47%)	271 (44.43%)	1.00 (ref)	<0.01	749 (17.84%)	62 (14.65%)	1.00 (ref)	0.095
Married or cohabiting	2525 (34.88%)	253 (41.48%)	1.46 (1.219, 1.743)		2881 (68.64%)	304 (71.87%)	1.27 (0.966, 1.708)	
Divorced or separated	745 (10.29%)	83 (13.61%)	1.62 (1.246, 2.089)	<0.01	426 (10.15%)	42 (9.93%)	1.19 (0.787, 1.788)	0.40
Widowed	26 (0.36%)	3 (0.48%)	1.68 (0.399, 4.806)	0.40	141 (3.37%)	15 (3.55%)	1.29 (0.688, 2.265)	0.41
Tuberculosis (%)								
Yes	103 (1.42%)	13 (2.13%)	1.00 (ref)	0.17	102 (2.43%)	13 (3.07%)	1.00 (ref)	0.42
No	7136 (98.58%)	597 (97.87%)	0.66 (0.384, 1.245)		4095 (97.57%)	410 (96.83%)	0.79 (0.454, 1.479)	
Symptom (%)								
Yes	1143 (15.79%)	107 (17.54%)	1.00 (ref)	0.26	625 (14.89%)	87 (20.57%)	1.00 (ref)	0.002
No	6096 (84.21%)	503 (81.46%)	0.88 (0.712, 1.101)		3572 (85.11%)	336 (79.43%)	0.68 (0.528, 0.873)	
Clinical stage (%)								
Clinical I	2783 (38.44%)	219 (35.90%)	1.00 (ref)		1670 (39.79%)	150 (35.46%)	1.00 (ref)	
Clinical II	2059 (28.44%)	175 (28.69%)	1.08 (0.878, 1.327)	0.47	1110 (26.45%)	115 (27.19%)	1.15 (0.893, 1.486)	0.272
Clinical III	1201 (16.59%)	105 (17.21%)	1.11 (0.869, 1.412)	0.39	702 (16.73%)	81 (19.15%)	1.28 (0.963, 1.702)	0.084
Clinical IV	1196 (16.53%)	111 (18.20%)	1.17 (0.927, 1.493)	0.18	715 (17.03%)	77 (18.20%)	1.20 (0.895, 1.595)	0.218
Weight (kg)	65.57 ± 8.60	64.99 ± 8.39	0.99 (0.982, 1.001)	0.11	63.18 ± 9.02	63.69 ± 9.21	1.00 (0.995, 1.017)	0.272
Height (cm)	172.20 ± 4.61	171.88 ± 4.30	0.98 (0.967, 1.002)	0.10	168.26 ± 6.84	168.91 ± 6.36	1.01 (0.999,1.030)	0.06
Leucocyte	5.58 ± 1.80	5.25 ± 1.74	0.89 (0.850, 0.940)	<0.01	5.10 ± 1.84	4.89 ± 1.88	0.94 (0.886, 0.995)	0.03
Platelet	193.54 ± 58.29	173.43 ± 59.34	0.993 (0.992, 0.995)	<0.01	180.18 ± 61.71	156.84 ± 61.79	0.993 (0.991, 0.995)	<0.01
Hemoglobin	146.89 ± 17.11	144.61 ± 18.49	0.99 (0.988, 0.997)	0.002	135.28 ± 20.85	135.07 ± 20.51	1.00 (0.995, 1.004)	0.84
Blood Creatinine	72.97 ± 13.15	70.81 ± 12.50	0.987 (0.980, 0.993)	<0.01	66.98 ± 16.08	66.96 ± 16.29	1.00 (0.994, 1.001)	0.98
Total cholesterol	4.01 ± 0.77	3.95 ± 0.72	0.89 (0.803, 1.000)	0.52	4.10 ± 0.79	4.02 ± 0.72	0.88 (0.774, 1.005)	0.06
Triglyceride	1.43 ± 0.81	1.43 ± 0.80	1.00 (0.896, 1.100)	0.95	1.50 ± 0.79	1.45 ± 0.68	0.894 (0.771, 1.024)	0.12
Glucose	5.58 ± 1.14	5.60 ± 1.13	1.01 (0.941, 1.083)	0.71	5.69 ± 1.41	5.60 ± 1.37	0.95 (0.878,1.028)	0.24
ALT	27.64 ± 18.03	33.67 ± 21.34	1.014 (1.011, 1.019)	<0.01	26.27 ± 17.59	32.59 ± 20.68	1.016 (1.011, 1.021)	<0.01
AST	24.74 ± 10.46	29.64 ± 14.24	1.031 (1.025, 1.037)	<0.01	26.32 ± 11.74	32.63 ± 16.47	1.03 (1.024, 1.037)	<0.01
Total bilirubin	12.45 ± 6.16	13.15 ± 7.01	1.016 (1.003, 1.028)	0.007	11.38 ± 5.79	13.16 ± 9.02	1.035 (1.022, 1.048)	<0.01
Treatment (%)								
Yes	1265 (17.47%)	128 (20.98%)	1.00 (ref)	0.030	853 (20.32%)	91 (21.51%)	1.00 (ref)	0.56
No	5974 (82.53%)	482 (79.02%)	0.79 (0.652, 0.981)		3344 (79.68%)	332 (78.49%)	0.93 (0.732, 1.193)	
Geographical setting (%)								
Jiangsu province	7025 (97.04%)	598 (98.03%)	1.00 (ref)		4094 (97.55%)	415 (98.11%)	1.00 (ref)	
Others	214 (2.96%)	12 (1.97%)	0.659 (0.347, 1.134)	0.16	103 (2.45%)	8 (1.89%)	0.766 (0.341, 1.487)	0.472

**Table 3:** Original data and balanced data.

Dataset	Minority class	Majority class	Samples in total	Imbalance rate
Heter (original)	423	4197	4620	1 : 9.922
Heter (SMOTE)	4158	4197	8385	1 : 1.009
Homo (original)	610	7239	7849	1 : 11.867
Homo (SMOTE)	7243	7239	14482	1 : 0.999

**Table 4:** Results of classification algorithms in homosexual group.

Testing criteria	DT	RF	AB	XGB
Confusion matrix	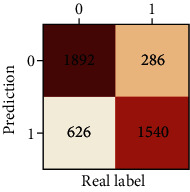	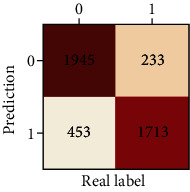	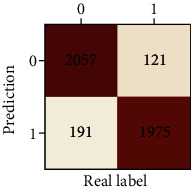	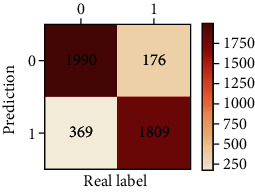
Accuracy	0.779	0.844	0,928	0.875
Precision	0.750	0.804	0.915	0.844
Recall	0.839	0.910	0.944	0.919
*F*-1	0.792	0.854	0.930	0.880
AUC	0.805	0.921	0.982	0.944

**Table 5:** Results of classification algorithms in heterosexual group.

Testing criteria	DT	RF	AB	XGB
Confusion matrix	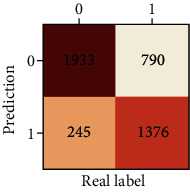	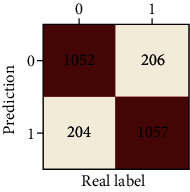	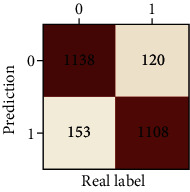	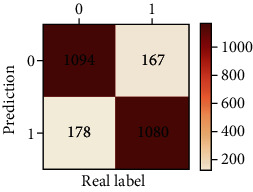
Accuracy	0.762	0.837	0.892	0.863
Precision	0.710	0.838	0.881	0.860
Recall	0.888	0.836	0.905	0.868
*F*-1	0.789	0.837	0.893	0.864
AUC	0.794	0.911	0.957	0.923

## Data Availability

The data used to support the findings of this study are available from the corresponding author upon request.

## References

[1] Liu H., Zhao M., Ren J. (2018). Identifying factors associated with depression among men living with HIV/AIDS and undergoing antiretroviral therapy: a cross-sectional study in Heilongjiang, China. *Health Qual Life Outcomes*.

[2] da Silva L. R., Araújo E. T. H., Carvalho M. L. (2018). Epidemiological situation of acquired immunodeficiency syndrome (AIDS)-related mortality in a municipality in northeastern Brazil. A retrospective cross-sectional study. *Sao Paulo Medical Journal*.

[3] Li Z., Teng Z., Miao H. (2017). Modeling and control for HIV/AIDS transmission in China based on data from 2004 to 2016. *Computational and Mathematical Methods in Medicine*.

[4] Nelson N. P., Easterbrook P. J., McMahon B. J. (2016). Epidemiology of hepatitis B virus infection and impact of vaccination on disease. *Clinics in Liver Disease*.

[5] Schweitzer A., Horn J., Mikolajczyk R. T., Krause G., Ott J. J. (2015). Estimations of worldwide prevalence of chronic hepatitis B virus infection: a systematic review of data published between 1965 and 2013. *Lancet*.

[6] Sarmati L., Malagnino V. (2019). HBV infection in HIV-driven immune suppression. *Viruses*.

[7] Brites-Alves C., Luz E., Netto E. M. (2018). Immune activation, proinflammatory cytokines, and conventional risks for cardiovascular disease in HIV patients: a case-control study in Bahia, Brazil. *Frontiers in Immunology*.

[8] Su M., Liao L., Xing H. (2018). Characteristics of HBV infection in 705 HIV-infected patients under lamivudine-based antiretroviral treatment from three regions in China. *Infection and Drug Resistance*.

[9] Xue M., Su Y., Li C., Wang S., Yao H. (2020). Identification of potential type II diabetes in a large-scale Chinese population using a systematic machine learning framework. *Journal of Diabetes Research*.

[10] Liang H., Tsui B. Y., Ni H. (2019). Evaluation and accurate diagnoses of pediatric diseases using artificial intelligence. *Nature Medicine*.

[11] Deniz E., Şengür A., Kadiroğlu Z., Guo Y., Bajaj V., Budak Ü. (2018). Transfer learning based histopathologic image classification for breast cancer detection. *Health Information Science and Systems*.

[12] Ashour A. S., Hawas A. R., Guo Y. (2018). Comparative study of multiclass classification methods on light microscopic images for hepatic schistosomiasis fibrosis diagnosis. *Health Information Science and Systems*.

[13] Price J. C., Seaberg E. C., Badri S., Witt M. D., D’Acunto K., Thio C. L. (2012). HIV monoinfection is associated with increased aspartate aminotransferase-to-platelet ratio index, a surrogate marker for hepatic fibrosis. *Journal of Infectious Diseases*.

[14] Demosthenes J. P., Sachithanandham J., Fletcher G. J. (2019). Characteristics of treatment-naïve HBV-infected individuals with HIV-1 coinfection: a cross-sectional study from South India. *Indian Journal of Medical Microbiology*.

[15] Sterling R. K., Wahed A. S., King W. C. (2019). Spectrum of liver disease in hepatitis B virus (HBV) patients co-infected with human immunodeficiency virus (HIV): results of the HBV-HIV cohort study. *American Journal of Gastroenterology*.

[16] Idoko J., Meloni S., Muazu M. (2009). Impact of hepatitis B virus infection on human immunodeficiency virus response to antiretroviral therapy in Nigeria. *Clinical Infectious Diseases*.

[17] Guha S. K., Sarkar J., Saha D. (2016). Baseline characteristics of HIV & hepatitis B virus (HIV/HBV) co-infected patients from Kolkata, India. *Indian Journal of Medical Research*.

[18] Konopnicki D., Mocroft A., de Wit S. (2005). Hepatitis B and HIV: prevalence, AIDS progression, response to highly active antiretroviral therapy and increased mortality in the EuroSIDA cohort. *AIDS*.

[19] Wandeler G., Gsponer T., Bihl F. (2013). Hepatitis B virus infection is associated with impaired immunological recovery during antiretroviral therapy in the Swiss HIV cohort study. *Journal of Infectious Diseases*.

[20] Tian X., Chong Y., Huang Y. (2019). Using machine learning algorithms to predict hepatitis B surface antigen seroclearance. *Computational and Mathematical Methods in Medicine*.

[21] Kammerer J. S., McNabb S. J. N., Becerra J. E. (2005). Tuberculosis transmission in nontraditional settings: a decision-tree approach. *American Journal of Preventive Medicine*.

[22] Maniruzzaman M., Rahman M. J., Al-MehediHasan M. (2018). Accurate diabetes risk stratification using machine learning: role of missing value and outliers. *Journal of Medical Systems*.

[23] Austin P. C., Tu J. V. (2004). Automated variable selection methods for logistic regression produced unstable models for predicting acute myocardial infarction mortality. *Journal of Clinical Epidemiology*.

[24] Shrivastava V. K., Londhe N. D., Sonawane R. S., Suri J. S. (2017). A novel and robust Bayesian approach for segmentation of psoriasis lesions and its risk stratification. *Computer Methods and Programs in Biomedicine*.

[25] Lee B. J., Ku B., Nam J., Pham D. D., Kim J. Y. (2014). Prediction of fasting plasma glucose status using anthropometric measures for diagnosing type 2 diabetes. *IEEE Journal of Biomedical and Health Informatics*.

[26] Li C. P., Zhi X. Y., Ma J. (2012). Performance comparison between logistic regression, decision trees, and multilayer perceptron in predicting peripheral neuropathy in type 2 diabetes mellitus. *Chinese Medical Journal*.

[27] Lavrac N. (1999). Selected techniques for data mining in medicine. *Artificial Intelligence in Medicine*.

[28] Harrell F. J., Califf R. M., Pryor D. B., Lee K. L., Rosati R. A. (1982). Evaluating the yield of medical tests. *JAMA: The Journal of the American Medical Association*.

[29] Terrault N. A., Lok A. S. F., McMahon B. J. (2018). Update on prevention, diagnosis, and treatment of chronic hepatitis B: AASLD 2018 hepatitis B guidance. *Hepatology*.

[30] Deng H., Deng X., Liu Y. (2018). Naturally occurring antiviral drug resistance in HIV patients who are mono-infected or co-infected with HBV or HCV in China. *Journal of Medical Virology*.

[31] Xie J., Han Y., Qiu Z. (2016). Prevalence of hepatitis B and C viruses in HIV-positive patients in China: a cross-sectional study. *Journal of the International AIDS Society*.

[32] Mocroft A., Kirk O., Barton S. E. (1999). Anaemia is an independent predictive marker for clinical prognosis in HIV-infected patients from across Europe. EuroSIDA study group. *AIDS*.

[33] Ranin J., Salemovic D., Brmbolic B. (2018). Comparison of demographic, epidemiological, immunological, and clinical characteristics of patients with HIV mono-infection versus patients co-infected with HCV or/and HBV: a Serbian cohort study. *Current HIV Research*.

[34] Thio C. L., Smeaton L., Saulynas M. (2013). Characterization of HIV-HBV coinfection in a multinational HIV-infected cohort. *AIDS*.

[35] Sterling R. K., Lissen E., Clumeck N. (2006). Development of a simple noninvasive index to predict significant fibrosis in patients with HIV/HCV coinfection. *Hepatology*.

[36] Jain M. K., Seremba E., Bhore R. (2012). Change in fibrosis score as a predictor of mortality among HIV-infected patients with viral hepatitis. *AIDS Patient Care and STDs*.

[37] Rotman Y., Brown T. A., Hoofnagle J. H. (2009). Evaluation of the patient with hepatitis B. *Hepatology*.

[38] Evans R. H., Scadden D. T. (2000). Haematological aspects of HIV infection. *Baillière's Best Practice & Research. Clinical Haematology*.

[39] Anabire N. G., Tetteh W. J., Obiri-Yaboah D. (2019). Evaluation of hepatic and kidney dysfunction among newly diagnosed HIV patients with viral hepatitis infection in Cape Coast, Ghana. *BMC Research Notes*.

